# The Early Diagnosis of Carcinoma Ventriculi

**Published:** 1908-05-09

**Authors:** 


### Medicine.

### THE EARLY DIAGNOSIS OF CARCINOMA VENTRICULI.

There is good reason to suppose that cancer of the stomach is a disease which could be cured by excision of the growth, if only the diagnosis could be made at an earlier stage than is usually the case at present. Every method which may assist in the diagnosis of the lesion when the symptoms differ little, if at all, from those of dyspepsia cannot but be valuable.

There is, unfortunately, no pathognomonic sign of early carcinoma ventriculi. The most that can be done is to apply several different clinical tests in a given case, and then to be guided by the results according as the majority of them are either positive or negative. If all the tests are negative the case would naturally require further watching, and repetition of these tests after not too long an interval. It should be always kept in mind that every patient who has symptoms of dyspepsia for the first time when the cancer age has been reached may have a gastric carcinoma, and that it is one's duty to take every possible step to arrive at a correct diagnosis at the earliest moment, and before a decided lump can be felt. It is better worth while to perform laparotomy and yet find no carcinoma than to postpone it simply because the diagnosis is not absolutely certain, and then find that the patient is beyond the power of operative skill to cure.

Among other clinical laboratory tests which have been used to help in the diagnosis of cancer of the stomach are three or four of real importance. Much stress has been laid upon the absence of free hydrochloric acid. The test is so well known that there is no need to say much about it here; but it is always important to keep in mind the fact that its clinical value is distinctly limited.

In the first place, even persons in robust health have no free hydrochloric acid in their gastric contents for a certain length of time after a meal. The time that must elapse between the eating of a meal and the appearance of free hydrochloric acid in the stomach will vary, in a rough measure, inversely as the amount of proteid in the meal. It is on this account that test meals of known composition are used; and whenever a specimen of vomit is sent to the laboratory for analysis it is very important to state clearly both at what time the last meal was eaten and also the quantities of the different food-stuffs that were then taken. We know of no clearer exposition of this subject than that given in French's Medical Laboratory Methods and Tests.

Secondly, there are a very large number of different conditions of ill-health in which there is a deficiency of the hydrochloric acid in the gastric juice. Absence of free hydrochloric acid at a time when it ought to be present therefore by no means proves the presence of a gastric carcinoma. It is only when the diagnosis has already been narrowed down by other means to such a degree that a gastric lesion of some kind is certain, and when the only doubt is as to whether the stomach trouble is simple or malignant, that the deficiency of the hydrochloric acid is an indication in favour of the latter, though by no means a certain one even then.

Moreover, in the earlier stages of gastric carcinoma hydrochloric acid may be present in abundance. It is only in cases no longer early that the hydrochloric acid is decidedly deficient. Although, therefore, deficiency of free hydrochloric acid under certain circumstances is a very valuable support to a diagnosis of malignant disease of the stomach, the converse is not true. One cannot exclude gastric carcinoma, especially early carcinoma, merely upon the ground that the vomit contains a normal quantity of free hydrochloric acid.

Salomon's test consists in washing out the stomach at night, some time after the last meal of the day, until the normal saline solution with which the lavage is performed comes back perfectly clear. Next morning, before any food is taken, the gastric lavage is repeated with one pint of normal saline solution. The latter is run in and out of the stomach through the tube several times, and when it is finally recovered it is tested quantitatively for albumen by Esbach's albuminometer. If it contains 20 milligrams of albumen per 100 cubic centimetres of fluid it is held that support is given to a diagnosis of carcinoma. The test is by no means pathognomonic, as Romano has pointed out, for simple ulcer and even certain conditions of acute gastritis may give rise to a secretion as albuminous as this; but taken in conjunction with other things, the test is of some assistance.

Salkowski's test consists in adding a 5-per-cent. solution of phosphoric acid to the patient's urine, centrifugalising, and testing for albumose. Albumosuria may, of course, occur in other conditions besides gastric carcinoma; but if the diagnosis is already suspected to be that of malignant disease of the stomach, and there is albumosuria, support is afforded to that diagnosis. If, on the other hand, there is no albumosuria, the condition may still be one of malignant disease of the stomach, for, according to von Adler, it is only in about two-thirds of these cases that Salkowski's test is positive.

Von Adler has devised a much more trustworthy test, which consists in excluding meat from the patient's dietary for a few days, and then examining the fæces for traces of blood. It is necessary, of course, to exclude all likelihood of there being intestinal hæmorrhage from colitis or hæmorrhoids, or the like, before the finding of traces of blood in the meat-free fæces can be held to point to ulceration of the stomach or duodenum. The fæces are mixed with a moderate quantity of normal saline solution acidulated with a few drops of glacial acetic acid; after standing for some time the supernatant fluid is decanted off or separated by filtration, and the ordinary tests for blood are applied to it. The guaiacum and ozonic ether test may be employed as for urine, or, better still, the benzidine test may be used. A hot solution of benzidine in absolute alcohol is poured into the fluid to be tested, and a few drops of oxygen water are added; if blood is present a blue-green colour is obtained. Another way of performing the benzidine test is that devised by Einhorn. Strips of absorbent paper are steeped in glacial acetic acid in which benzidine has been dissolved to saturation. When well soaked they are taken out and dried. The benzidine test-papers so prepared must be kept in a carefully stoppered jar, and in using them it is important not to let them come into contact with organic matter, such as the hand. They must be held, not in the fingers, but in forceps, and dipped into the fluid to be tested. Over the paper thus moistened with the specimen supposed to contain blood a few drops of hydrogen peroxide are poured, and if blood is present the colour of the strip will change first to green and then to blue within a minute or two. The test is, of course, quite as applicable to urine and to vomit as it is to fæcal extracts.

If then, in a given case in which, although no lump can be felt, gastric carcinoma is suspected from the symptoms, it is found that the three last-mentioned tests are positive, even though there be abundance of free hydrochloric acid in the gastric contents, and still more so if that hydrochloric acid be deficient, there is considerable ground for a diagnosis of early carcinoma. It is all-important that these tests should be put to full account in the hope that the lesions may some day be detected earlier and in a more curable condition than is now the case. They are far from perfect, but meanwhile there are none better.

